# Ultrastructural and immunocytochemical evidence for the reorganisation of the milk fat globule membrane after secretion

**DOI:** 10.1007/s00441-016-2505-8

**Published:** 2016-09-27

**Authors:** F. B. Peter Wooding, Ian H. Mather

**Affiliations:** 1 Physiology Neuroscience and Development Department, Cambridge University, Cambridge, CB2 3EG UK; 2 Department of Animal and Avian Sciences, University of Maryland, College Park, MD20742 USA

**Keywords:** Milk fat globule membrane, Ultrastructure, Immunocytochemistry, Paracrystalline arrays, Unit membrane

## Abstract

This paper reports a detailed ultrastructural and immunocytochemical investigation of the structure of the milk fat globule membrane (MFGM) in a variety of species. The process follows the same pattern in all mammals so far investigated. The initial (or primary) MFGM immediately on release from the mammary cell is a continuous unit membrane with a thin underlying layer of cytoplasmic origin and a monolayer of phospholipid separating it from the core lipid. This structure changes rapidly as the milk fat globule (MFG) moves into the alveolar lumen. The unit membrane plus the underlying layer of cytoplasm modifies drastically into discontinuous patches and networks. These are superimposed upon a continuous apparently structureless sheet of electron dense material stabilising the MFG and similar to that which bounded the lipid in the cell. The underlying layer of the patches increases in electron density and immunocytochemistry demonstrates localisation of MFGM proteins in this layer. In four species, the dense material shows ordered paracrystalline molecular arrays in section and en face views. All the arrays show the same basic pattern and unit size as determined by optical diffraction. Similar patches, networks and arrays are present on the surface of expressed MFG. Negative staining of lipid-extracted expressed MFGs shows similar patches and networks of membrane. These also occasionally show the crystalline arrays and label with MFGM protein antibodies. Similar networks and strands of plasma membrane on the MFG surface are shown by our CLSM examination of unfixed expressed MFG from mice genetically modified to express a fluorescent molecule as a normal plasma membrane constituent.

## Introduction

According to a recent comprehensive review (Lopez 2011: p. 398) the structure of the milk fat globule membrane (MFGM) is “still not known in detail and remains the least understood aspect of the milk fat globule (MFG)”.

The functional importance of the MFGM lies in its ability to stabilise the lipid droplets in the milk, facilitate the uptake of MFG components by the neonatal gut and to supply the developmental cues provided by the biochemical constituents of the membrane. There is increasing evidence (DeWettinck et al. 2008; Lopez 2011) for the important role of MFGM constituents in the development of the neonatal gut and immune system and in providing antiviral and antimicrobial protection. Longer term, the high glycerolipid content could make an important contribution to nervous system development.

The MFGM is also important commercially for its valuable contribution to texture and flavour (DeWettinck et al. 2008), given the widespread use of milk and milk derivatives in the food industry.

There are currently two very different interpretations of MFGM structure in the alveolus and in expressed milk. There is a general agreement that, immediately after secretion from the mammary cell into the alveolus, transmission electron microscopyy (TEM) shows that the MFGM is a continuous unit membrane separated from the core lipid by a 15- to 20-nm layer of cytoplasmic origin (Wooding 1971a, b; Mather and Keenan 1998; Vorbach et al. 2002).

Recent confocal laser scanning microscopy (CLSM) studies of unfixed isolated MFGs from bovine and human milk using lipid probes suggest that the continuous unit membrane persists in the alveolus and after release from the glands (Evers et al. 2008; Gallier et al. 2010, 2015; Lopez 2011; Zheng et al. 2013).

In contrast, most early (Dowben et al. 1967; Henson et al. 1971; Wooding 1971a, b; Berendsen and Blanchette-Mackie 1979) and more recent TEM results (Armand et al. 1996; Gallier et al. 2015) can be interpreted to show that there is a drastic rearrangement of the MFGM into discontinuous patches and strands.

This study was designed to distinguish between these two possibilities using a wide range of species and a variety of EM and CLSM techniques. It provides new information on the TEM structure of the MFGM and direct evidence for the reorganisation of the MFGM molecular structure after secretion.

## Materials and methods

### Electron microscopy

Mammary tissue from lactating goats, ewes, rats, mice, guinea pigs, fur seals, horses, Friesian or Jersey cows and wallabies was either excised immediately after death by barbiturate overdose and cut into small cubes in an aldehyde, dichromate-acrolein or osmium fixative, or fixed initially by perfusion with aldehyde via the mammary artery (cow, ewe, goat, guinea pig and horse). All the processing was carried out at room temperature. Expressed milk was added to an equal volume of fixative.

All animal work was carried out in accordance with the UK Animals (Scientific Procedures) Act 1986 and the Animal Care and Use Committee of the National Institutes of Health, U.S.A. The usual fixation was in 4 % glutaraldehyde in 0.1 M phosphate buffer, pH 7.2, containing 2 % sucrose, for 45 min. Alternatively, 1 % osmium tetroxide in 0.1 M veronal buffer, pH 7.2 or 1.5 % acrolein + 1 % potassium dichromate + 6 % sucrose in 0.1 M veronal buffer at 4 °C were used.

The tissue was washed briefly in buffer, postfixed first in 1 % osmium tetroxide in 0.1 M veronal buffer, pH 7.2, for 30 min, then in 5 % aqueous uranyl acetate for 2 h followed by ethanol dehydration and embedding in Araldite. Sections were cut on an LKB Ultrotome, stained with uranyl acetate and lead hydroxide and observed in an AEI EM6B electron microscope operated at 60 kV.

### Electron microscope immunocytochemistry

Tissue was fixed by immersion or perfusion in 4 % formaldehyde in 0.1 M phosphate buffer, pH 7.2, or in 4 % formaldehyde plus 1 % glutaraldehyde in 0.1 M phosphate buffer, pH 7.2. No osmium tetroxide was used. Dehydration and embedding was at room temperature for Araldite resin. Thin sections were cut from the Araldite blocks and picked up on 300-mesh nickel grids. Resin was removed from the araldite sections by floating for 15 s on a solution of one volume of sodium ethoxide (15 g of sodium hydroxide pellets dissolved in 15 mls of absolute alcohol) diluted with four volumes of distilled water, followed by alcohol and water washes.

For immunocytochemistry, the grids were floated overnight section side down on drops of antibody, washed, incubated with immunogold colloid (goat anti-rabbit G10 or 15 nm; Jackson Immunoresearch Labs, USA), washed with buffer and water, stained with uranyl acetate and lead solutions and examined in a Philips Electron microscope (EM).

Two primary monospecific affinity purified antibodies were used: rabbit anti-BTN (bovine whole molecule, 1:1000) (Banghart et al. 1998) and rabbit anti XOR (bovine whole molecule, 1:100) (Sullivan et al. 1982 for preparation of protein and Banghart et al. 1998 for the immunoblot). Both antibodies were affinity purified on the respective purified antigen.

Immunocytochemical controls, in which the primary antibody was omitted and replaced with buffer or a non-specific antibody at the same concentration, were carried out routinely, alongside the experimental samples. Controls showed an insignificant level of labelling.

### Electron microscope negative staining

Freshly expressed uncooled milk was fixed with an equal volume of 4 % glutaraldehyde fixative for 60 min. The fixed milk was spun at ∼1500 rpm for 1 min. An EM grid was touched to the surface of a drop of the cream on parafilm, the grid held horizontal for 1 min, then washed with water and dried. The grid was immersed in n-heptane for 5 min to remove triglyceride, dried and negatively stained with a drop of 2 % potassium phosphotungstate, the excess blotted off and the grid dried before examination in the EM. For immunocytochemistry, the heptane-extracted grid was rehydrated and incubated overnight on XOR antibody and the antigen localised with 15-nm gold colloid before negative staining.

## Optical diffraction

The micrographs were analysed using a benchtop Optical Diffractometer (Amos 2005) by courtesy of Drs Amos and Richardson, MRC Laboratory of Molecular Biology, Cambridge.

### CLSM of mouse MFGs

Milk was collected as described (Ogg et al. 2004) from three green fluorescent protein (GFP)-membrane transgenic *mT/mG* mice (Muzumdar et al. 2007) on the tenth day of lactation. Samples of whole milk were immediately incubated at room temperature with BODIPY 665 dye (ThermoFisherScientific) at a final concentration of 10 μM for 30 min. This dye stains the triglyceride core of the MFG. Drops of the stained milk were placed on Superfrost/Plus microscope slides, sealed under coverslips and examined with a ×60 oil immersion objective in an Olympus FluoView 1000 confocal microscope. The GFP and BODIPY 665 fluorophores were excited at 488 and 633 nm and emissions collected at 520 and 688 nm, respectively. Optical sections (0.5 μm) through a *z* depth of 10–12 μm were recorded as TIFF files and three-dimensional images reconstructed from the *z*-stacks with Imaris software (Bitplane).

## Results

**Fig. 1:**
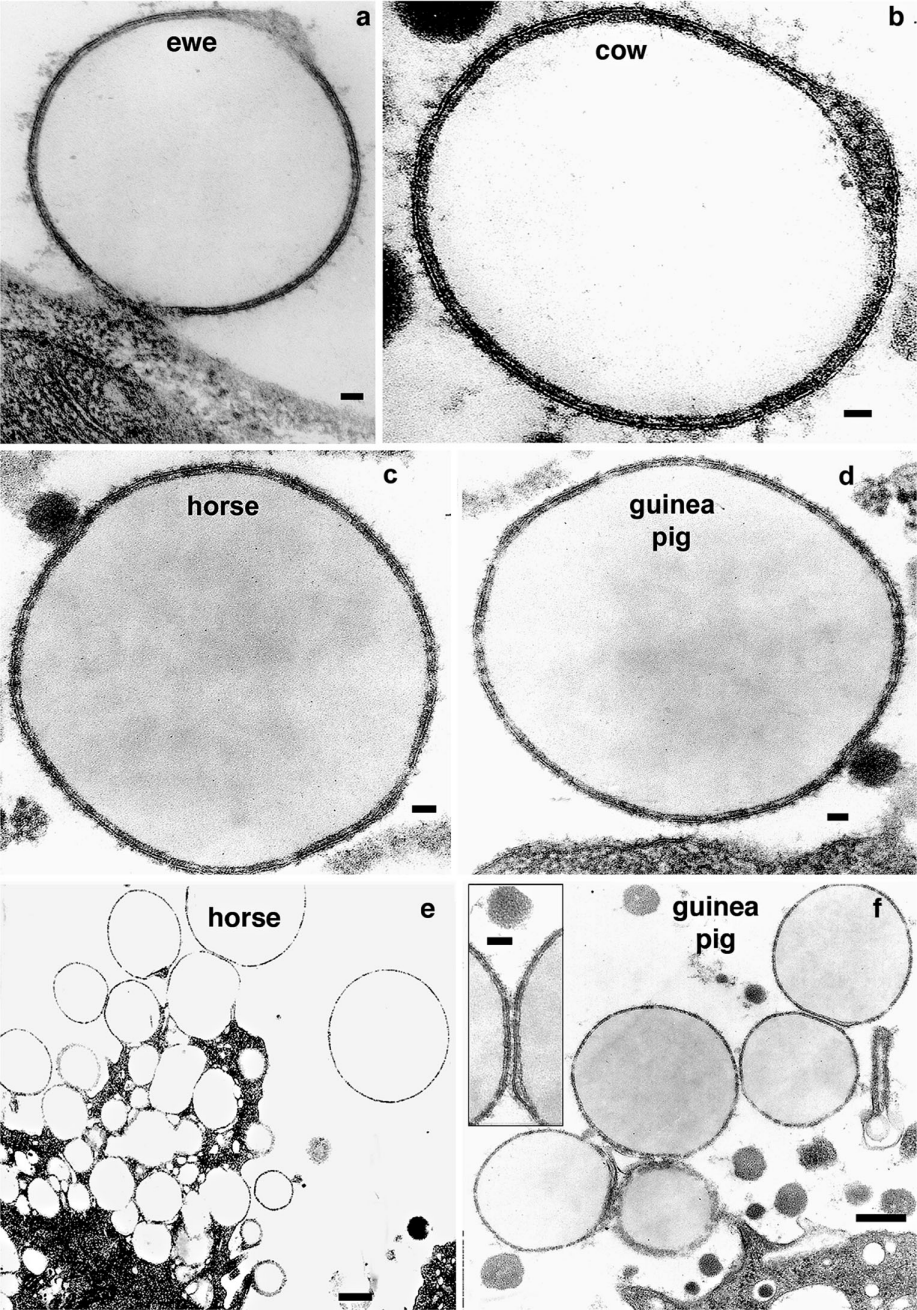
TEM of MFGs from a variety of genera just before (**a**) and immediately after (**b**–**f**) release from the mammary cell. Each is bounded by a continuous unit membrane with an inner thin weakly stained cytoplasmic layer enveloping the lipid droplet. All MFGs in (**e**) and (**f**) showed this PMFGM structure at sufficient magnification as illustrated in the *inset* in (**f**).* Bars* (**a**, **c**, **d**) 50 nm; (**b**) 75 nm; (**e**, **f**) 1 μm

**Fig. 2:**
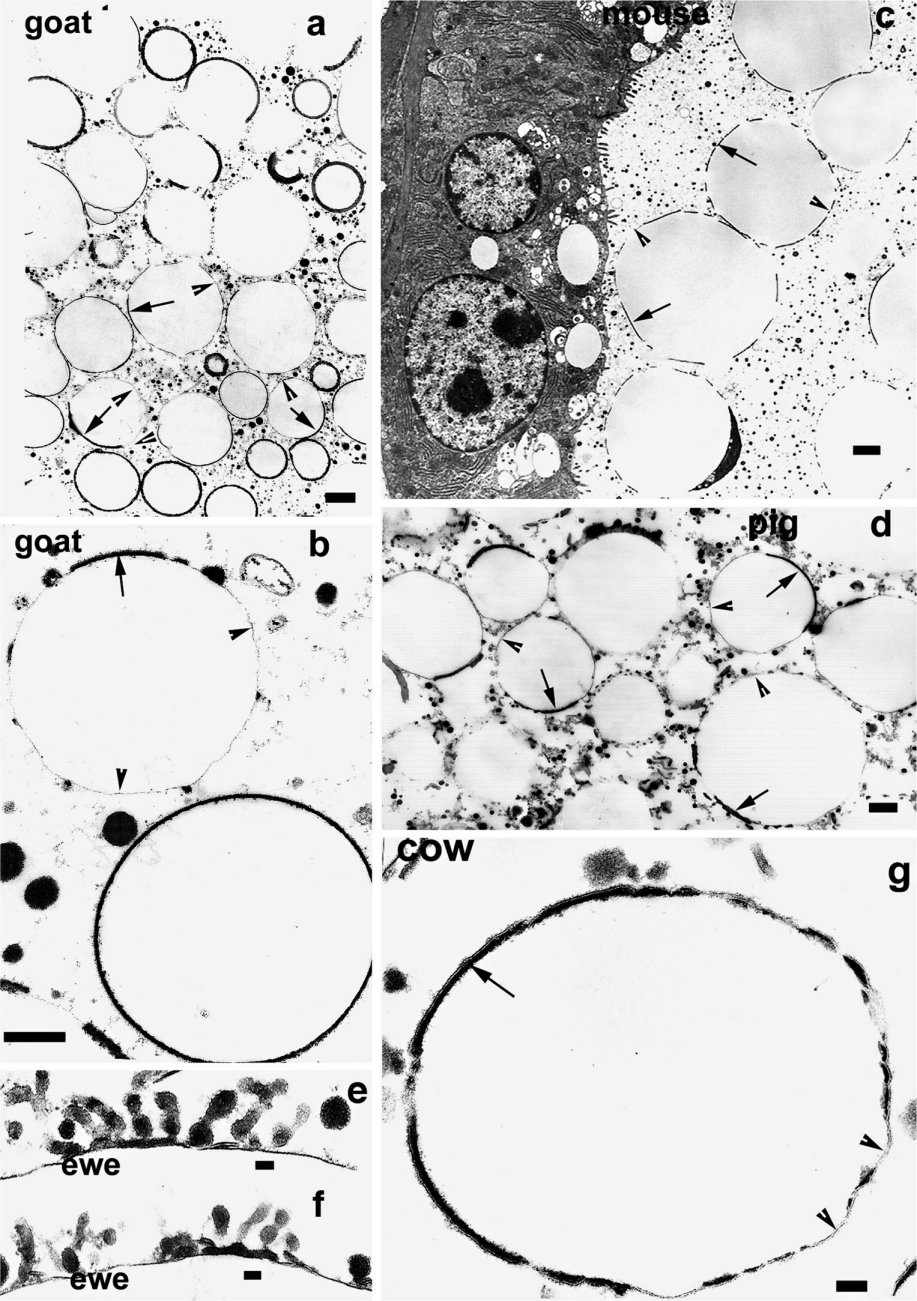
TEM of alveolar MFGs from a variety of genera. All the MFGs show a discontinuous RPMFGM consisting in a unit membrane overlying a cytoplasmic layer of considerably increased electron density (*arrows*). These RPMFGM patches and networks are supported by a continuous dense line (*arrowheads*) around each MFG. Examples of a loss of PMFGM by vesiculation (**e**, **f**) can be found in a small but significant number of MFG in all genera.* Bars* (**a**–**d**, **g**) 1 μm; (**e**, **f**) 50 nm

Under optimal perfusion conditions (Wooding 1977a, b) in ewe, cow, horse and guinea pig, only a few MFGs close or still tenuously attached to the secretory cells show a continuous unit membrane bounding an electron lucent space around the core lipid (Fig. 1a ewe; b cow; c, e horse; d, f guinea pig). Further into the alveolar lumen, MFGs show a variety of membrane structures most of which can be interpreted as resulting from a disruption and modification of the initial continuous membrane. Mammary tissue optimally fixed by immersion also shows these changes. These changes are coincident with or as a result of alterations in the interactions of the proteins in the material between the membrane and core lipid as indicated by an increase in EM density (Fig. 2a, b goat; c mouse; d pig; e, f ewe; g cow).

This process produces patches and networks on the surfaces of MFGs with a unit membrane overlying very electron-dense material seen on transverse sections in Fig. 2. These patches sit upon a continuous dense line, equivalent in EM appearance to the electron-dense line that was the lipid core boundary in the cytoplasm. This dense line forms the only continuous structure that stabilised the cytoplasmic lipid droplet in the cell and that now has a similar function in stabilising the secreted MFGs. The area covered by the modified membrane is very variable and different in different species. In sections such as those in Fig. 2, the membrane covers 30–50 % of the total area of the MFG in the alveolus in all the species we have so far examined.

**Fig. 3:**
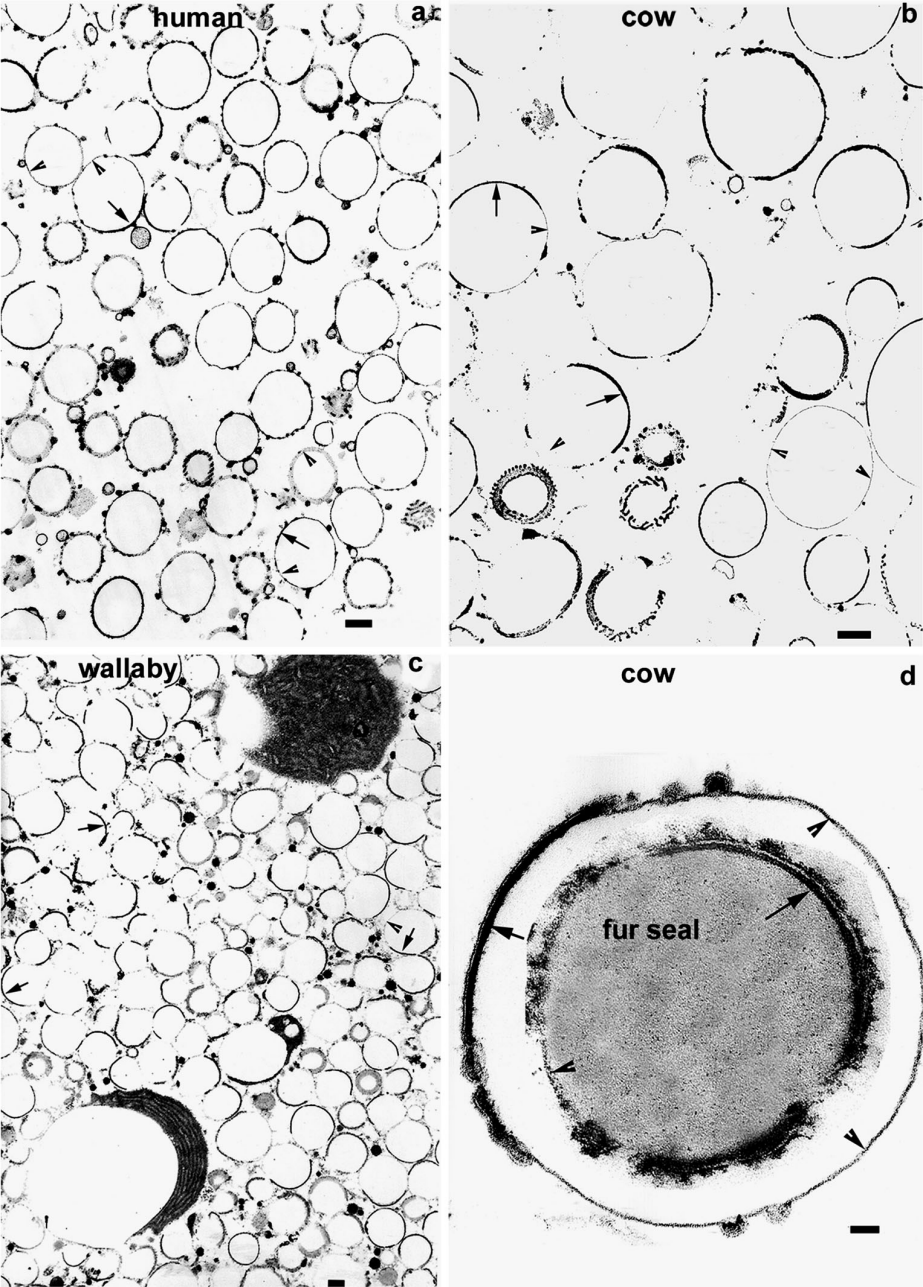
TEM of transverse sections of MFGs from expressed milk from a variety of genera demonstrating very similar RPMFGM discontinuities and increases in electron density of the cytoplasmic layer as in the alveolar MFGs. d Two examples at higher magnification illustrating the unit membrane of the discontinuous RPMFGM (*arrows*) covering the electron-dense layer and the single dense line of the SMFGM (*arrowheads*) enveloping the lipid core.* Bars* (**a**–**c**) 1 μm; (**d**) 50 nm

The dense line is comparatively thin and difficult to distinguish on low-power micrographs (Figs. 2a, c, 3a, c, arrowheads) but the uniformity of the circularity of the cross-sections of the MFG clearly indicate that it is present. At higher magnifications, it can be easily seen (Figs. 2b, d, g, 3d, arrowheads)

The EM evidence for loss of membrane by vesiculation or “blebbing” (Wooding 1971b; Fig. 2e, f) is found in all species examined. A second probably larger contributor to the drastic morphological change is a contraction of the membrane area presumably driven by an increase in protein molecular order in the dense material under the unit membrane.

For ease of discussion, the continuous unit membrane and its adherent layer will be referred to as the Primary MFGM (PMFGM) and the dense line as the Secondary MFGM (SMFGM) onto which patches and networks of Residual PMFGM (RPMFGM) are anchored by molecular interactions.

Expressed MFGs show a very similar discontinuous MFGM on TEM examination to those in the alveolus (Fig. 3a human; b, d cow; c wallaby; d inset, fur seal): a continuous dense line of SMFGM on which are superimposed RPMFGM patches of unit membrane plus underlying electron-dense material. No significant differences have been seen in the structure or percentage of RPMFGM as a result of expression from the gland and the stability of the membrane in expressed milk has been confirmed by biochemical studies (Baumrucker and Keenan 1973).

**Fig. 4:**
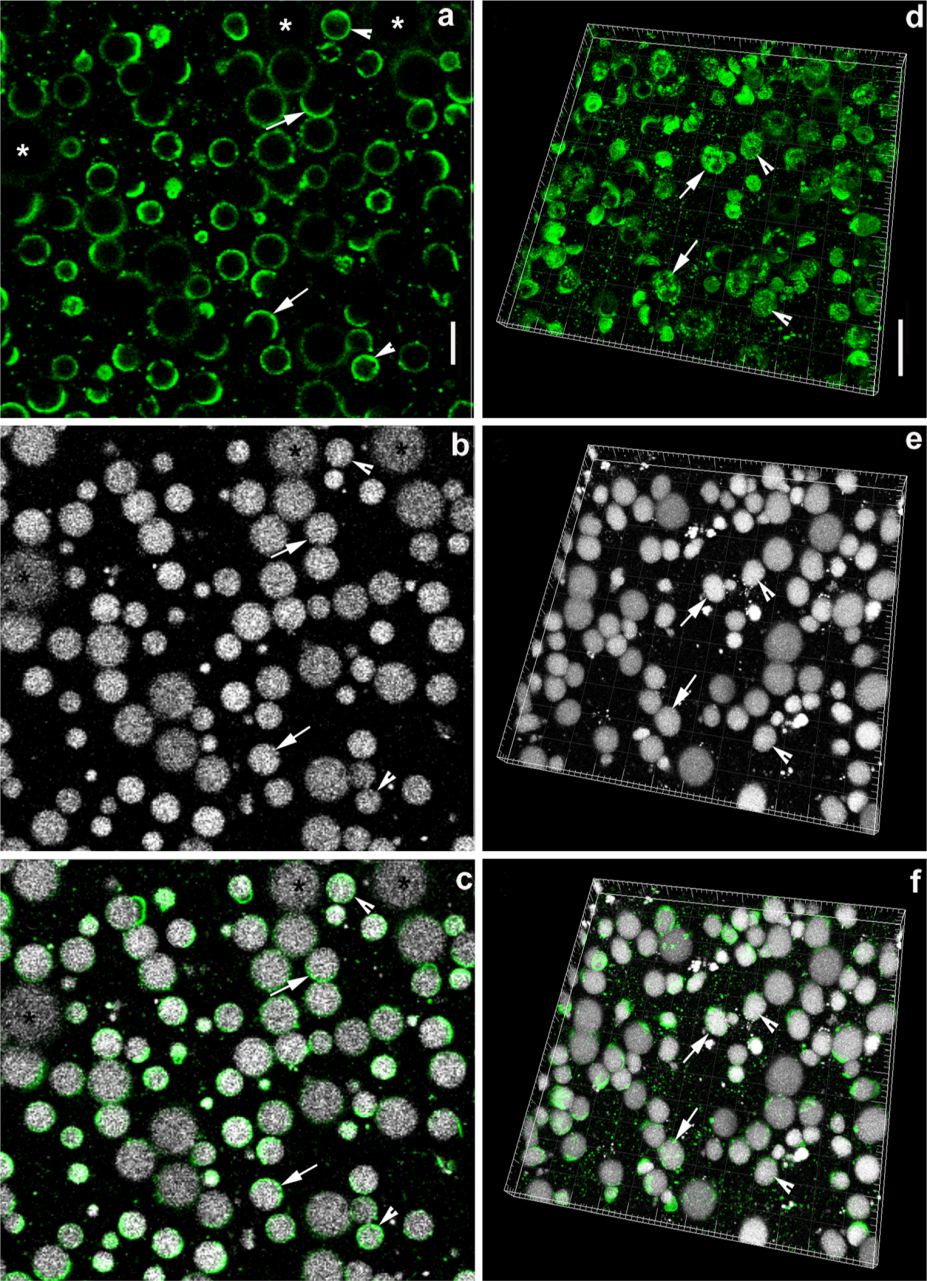
CLSM of MFGs in expressed milk from the GFP-membrane (*mT/mG*) mouse. **a**–**c** Two-dimensional CLSM images showing **a** GFP fluorescence and **b** BIODIPY 665 fluorescence of neutral lipid in the MFG; **c** overlay of (**a**) and (**b**). Note that the GFP-membrane fluorescence is associated with most of the MFG but in many cases is unevenly distributed on the surface:* asterisks* MFG with very little GFP fluorescence on this plane of section,* arrows* MFG with ∼50 % and* arrowheads* ∼100 %. **d**–**f** CLSM of MFGs in three-dimensional reconstructions showing uneven but global distribution of GFP-membrane fluorescence on MFG surfaces. Compare the GFP distribution on the MFG at the* arrows* with the TEM images of the MFG surface in Fig. 6. **e** BIODIPY 665 fluorescence of neutral lipid in the MFGs; **f** overlay of (**d**) and (**e**).* Bars* (**a**–**c**) 20 μm, (**d**–**f**) 20 μm

**Fig. 5:**
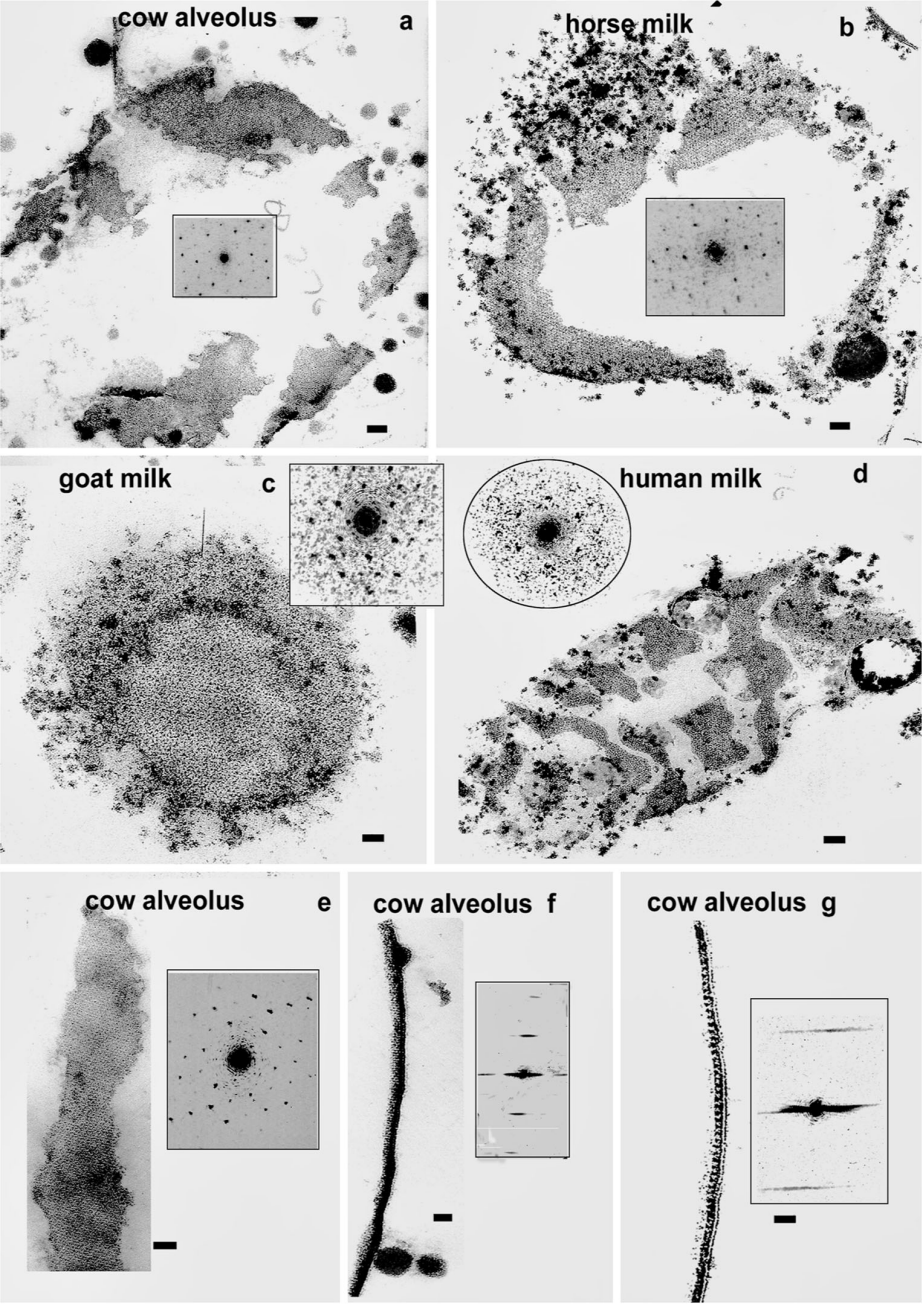
TEM of paracrystalline arrays in the RPMFGM. **a**–**e** En face views of the MFGM from a variety of genera all illustrating the paracrystalline arrays. **f**, **g** Transverse section views establish the exact location of the arrays in the dense layer underlying the unit membrane. The similarity of the organisation of each en face micrograph is emphasised by their optical diffraction patterns (*inset* on each) showing hexagonal patterning with equivalent unit sizes.** a**–**d**,** f**,** g** were all initially fixed in glutaraldehyde but the same organisation is found after initial dichromate–acrolein fixation of the cow mammary gland (**e**). Similar results were obtained with osmium (results not shown). Bars 20 nm

**Fig. 6:**
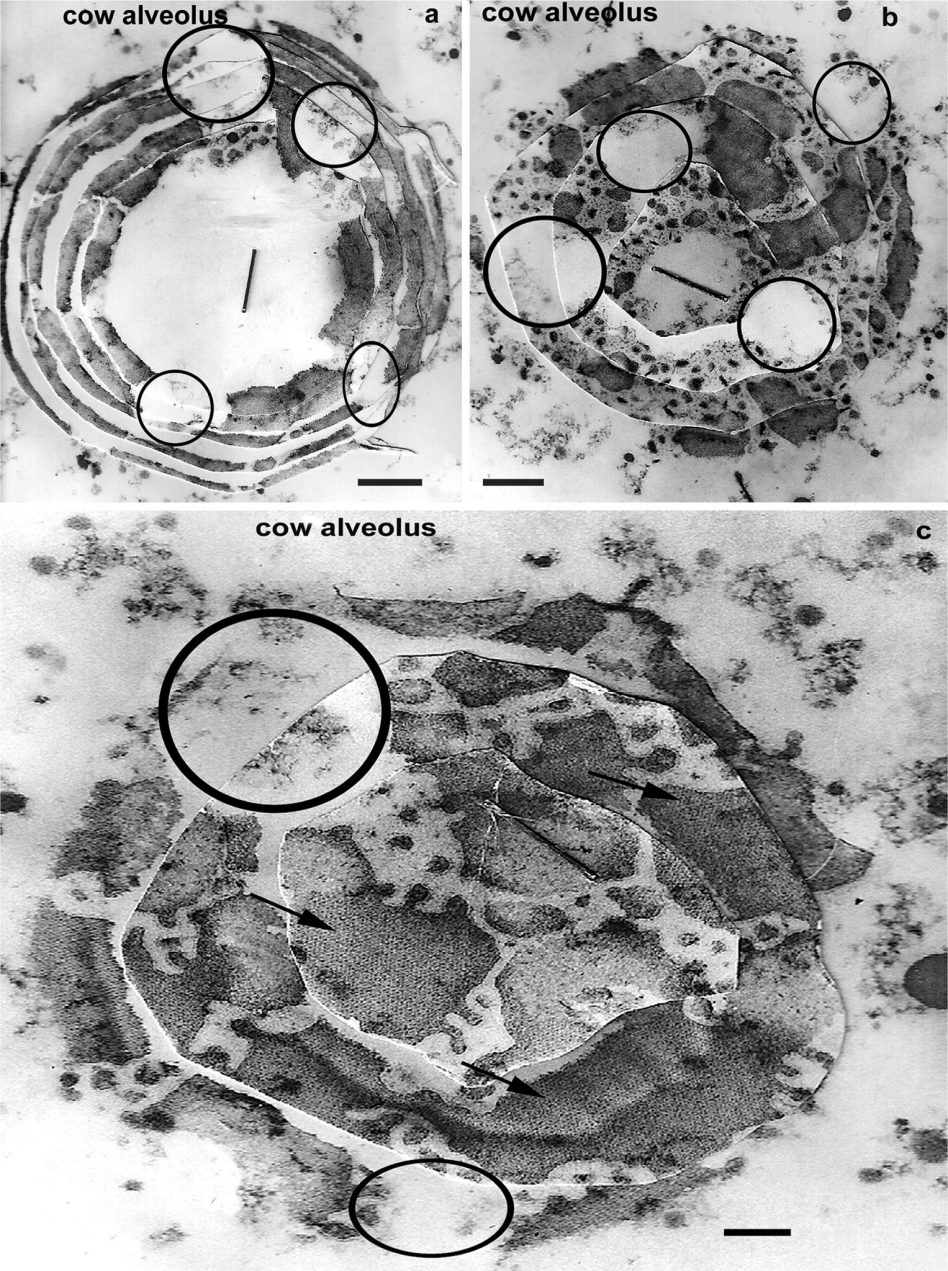
Reconstructions of RPMFGM structure from TEM serial sections of MFGs. The drastic modification of the originally continuous membrane produces a variety of structures but all show one or more small circular areas free of RPMFGM, similar in size to the areas found by CLSM; **c** also shows large areas (*arrows*) of paracrystalline organisation in the RPMFGM.* Bars* (**a**,** b**) 50 nm; (**c**) 100 nm

CLSM micrographs of transverse sections of unfixed expressed MFGs from transgenic mice that constitutively express green fluorescent protein (GFP) on their plasma membrane (Fig. 4a–c) clearly show an equivalent discontinuous distribution of the RPMFGM-like fluorescence on the contours of the MFGs on transverse sections (compare Figs. 2 and 3 with 4a–c). CLSM 3D reconstruction of MFGs demonstrate very similar patches and networks of membrane on their surfaces (Fig. 4d–f) equivalent to those on the electron micrographs on Figs. 5 and 6.

The close similarity in MFGM structure between all species examined is emphasized by the occurrence of a paracrystalline organisation in the dense material under the unit membrane in some alveolar luminal and expressed MFGs of four unrelated species. In Fig. 5, this can be seen in both en face (5a, c, e cow alveolar; b horse milk; c goat milk; d human milk) and transverse sections (f, g cow alveolar) of the MFG. The en face EM views in Figs. 5 and 6 clearly show the patch and network distribution of the RPMFGM that characteristically show a variety of edge structures including “finger fringed” edges (Figs. 5a, c, 6c; see also 8a, b, below). The evidence of a change to a greater molecular order in the dense material supports the idea of a post-secretion contraction of the original PMFGM producing the patches and networks.

The paracrystalline lattice is based on a similar hexagonal organisation and unit size with similar spacing in all species where it is found, as determined by optical diffraction techniques (Figs. 5a–g; see also 8c, below). It is not dependant on a primary glutaraldehyde fixation, as the same pattern is also produced in the cow MFGM by initial fixation in dichromate–acrolein (Fig. 5e) or in osmium tetroxide (results not shown).

TEM serial section reconstruction of cow alveolar MFGs in Fig. 6 show that small circular areas free of RPMFGM can often be clearly recognised.

**Fig. 7:**
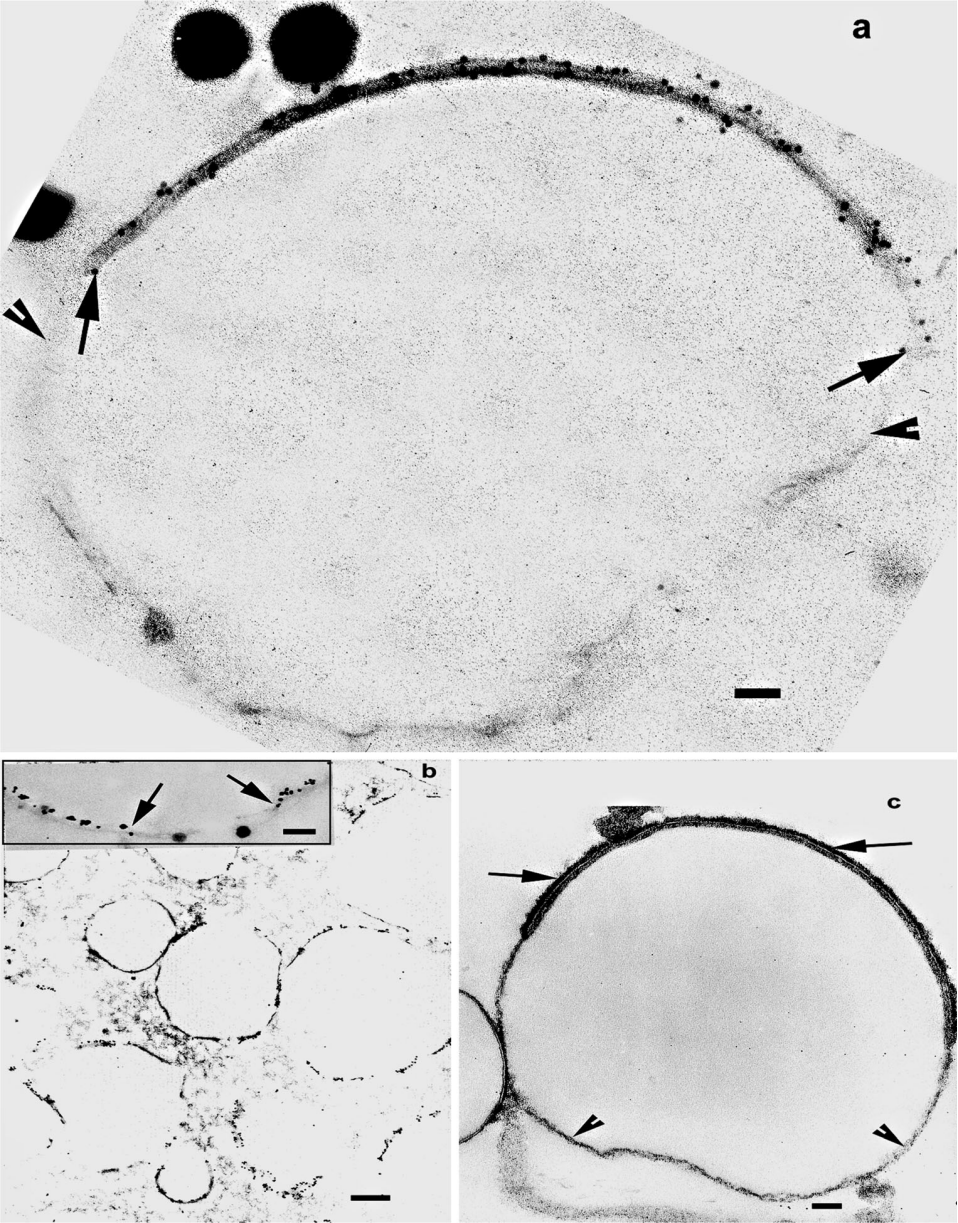
**a** TEM immunolocalisation of BTN on the cow RPMFGM (between *arrows*) but not on the dense line (between *arrowheads*) that is part of the SMFGM. **b** shows the discontinuous distribution of the BTN label on human MFGs, very similar to the discontinuous pattern of RPMFGM in Fig. 3a. The section staining is insufficient to clearly distinguish RPMFGM and SMFGM but at higher magnification in the *inset* the interruption in the gold colloid labelling is clear between the *arrows*. **c** From cow milk fixed with 500 ppm of Ruthenium red added to the aldehyde fixative. The glycocalyx is clearly shown (*arrows*) on the outside of the RPMFGM unit membrane but no label is apparent on the SMFGM (*arrowheads*).* Bars* (**a**,* inset* in** b**,** c**) 50 nm; (**b**) 1 μm

Immunocytochemical labelling of the sections with antibodies to MFGM proteins shows that only the dense material of the RPMFGM labels; the continuous dense line enveloping the whole MFG is unlabeled (Fig. 7a, b). Emphasizing the difference between the two areas of SMFGM, when Ruthenium red is used to probe for any glycocalyx on the outer surface of the MFG, again the label is only on the RPMFGM unit membrane area of the SMFGM (Fig. 7c).

**Fig. 8:**
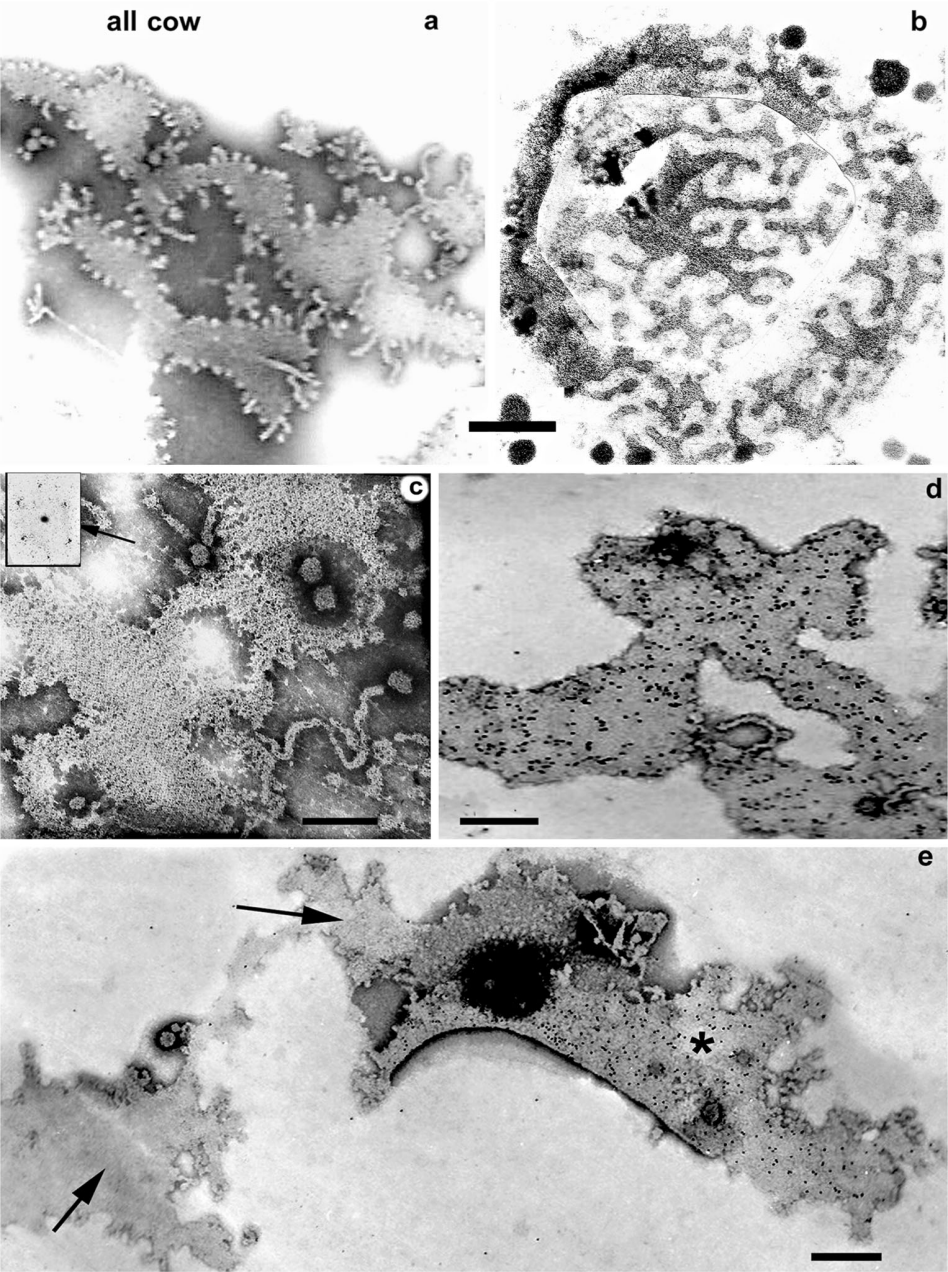
TEM of cow MFGM negatively stained with phosphotungstic acid. **a** Negatively stained lipid-extracted MFG demonstrating membrane sheets of a characteristic outline very similar to RPMFGM as seen en face on sectioned material in (**b**). **c** The negatively stained RPMFGM sheet shows a similar paracrystalline organisation to that found on the sectioned MFG. Inset at arrow; optical diffraction pattern. **d** TEM immunocytochemistry using 10-nm gold particles to demonstrate the distribution of XOR in the MFGM. Some but not all, of the negatively stained RPMFGM sheets on a grid can be immunolabeled with XOR (or BTN, results not shown) antibodies suggesting that only one side of the sheet labels. Where such membrane sheets are folded back, the 10-nm immunolabel is only seen on one side (compare the areas indicated by the *arrow* and *asterisk* in **e**) confirming this hypothesis. *Bars* 50 nm

The reality of this membrane structure is reinforced by TEM examination of negatively stained membrane from individual, fixed isolated expressed MFGs (Fig. 8a, c–e). Extraction of the core lipid from MFGs adherent to a carbon-coated EM grid and subsequent negative staining with phosphotungstic acid or uranyl acetate reveal membrane patches with finger fringing (Fig. 8a) equivalent to those demonstrated en face (Fig. 8b) on EM sections.

Prior incubation of the lipid extracted membrane patches with antibodies to MFGM proteins localised with gold colloid either show dense or no label (Fig. 8d, e), presumably depending on which side of the membrane patch was uppermost. This assumption is supported by the observation that where the membrane is partially folded back on itself during preparation, only one side of the fold is labelled (Fig. 8e).

Occasionally the negatively stained membrane patches show a similar paracrystalline organisation to that demonstrated on the sections (Fig. 8c) and OD analysis (inset, arrow) shows a similar hexagonal pattern and unit size to the section material.

## Discussion

This paper establishes for the first time the uniformity of the detailed structural changes characteristic of the MFGM after secretion from the mammary cell in a variety of mammalian genera using a range of TEM and light microscope techniques. This confirms and considerably extends the primary author’s original studies (Wooding 1971a, b, 1977a, b). The continuity of the unit membrane that packages the cytoplasmic lipid droplet is necessary to permit repeated release of the lipid without damaging the mammary cell. The ingenious solution of collaboration between Golgi vesicles and plasmalemma (Wooding and Sargeant 2015) provides the considerable amount of packaging membrane (Mather 2011) required for MFGM formation.

Once the MFG is released from the cell, the continuous unit membrane is apparently unnecessary and some may be lost by vesiculation (“blebbing”) and the remainder considerably reduced in area by interactions between the MFGM proteins being sufficiently strong to cause contraction of the area of the unit membrane with its underlying protein layer. This produces the isolated finger fringed patches and networks of RPFGM on the SMFGM. This process occurs rapidly and describes 90 % of the alveolar MFGs. Stability of the MFG is then dependant solely on the SMFGM that forms a continuous envelope equivalent to that which originally bounded the lipid in the cytoplasm. It appears as a continuous dense line on EM sections and is stable enough to survive expression from the mammary gland and storage.

This process of alveolar PMFGM reorganisation occurs in all species so far examined with optimally fixed EM samples and the uniformity of the modifications are exemplified by the equivalence of the paracrystalline structures in the dense material underneath the unit membrane in cow, goat, horse and human. The first report and illustration of paracrystalline areas in finger-fringed PMFGM patches was described in cow sectioned material (Wooding 1977a: figs 34, 35; Wooding 1977b). The reality of such membrane structures has subsequently been confirmed by Bucheims freeze-fracture studies (Bucheim 1982, 1986; Schmidt and Buchheim 1992) showing very similar images of equivalent unit size. The freeze-fracture images were produced from unfixed milk with no glycerol added by ultra-rapid cryofixation (Buchheim 1982), yet the micrographs show equivalent RPMFGM patches including some with paracrystalline arrays. These are very similar to those shown in this paper by conventional TEM processing. We consider Buchheim’s results with freeze-fracture more reliable than those of Robenek et al. (2006) whose localisations and images do not agree with other results in the literature.

There are five papers (Dowben et al. 1967, cow; Henson et al. 1971, cow; Berendsen and Blanchette-Mackie 1979, rat; Armand et al. 1996 and Gallier et al. 2015, both human) with TEM micrographs that corroborate the discontinuities in MFG in expressed milk. There are also two papers (Freudenstein et al. 1979, human; Vorbach et al. 2002, mouse) using TEM that claim that in freshly expressed milk all or most of the MFG are covered with PMFGM. Unfortunately, neither of these two papers has micrographs showing several MFG at sufficient resolution to distinguish RPMFGM from SMFGM, plus higher magnification of detail with the unit membrane resolved such as in Figs. 2 and 3 in this paper. This is necessary to allow independant judgement of claims of membrane continuity.

In this paper, immunocytochemical results from sections confirms that MFGM proteins including BTN and XOR are present in the dense material and this is reinforced by the MFGM antibody–gold colloid labelling of the negatively stained RPMFGM areas.

Most of the results above are EM section-based and all require prior sample fixation with chemicals. We believe the structure and changes in the MFGM illustrated above are an accurate representation of the normal process in alveolar and expressed MFGs. Our conclusions are based on (1) perfusion of tissue providing simultaneous rapid optimal fixation throughout, producing spherical lipid droplets with unit membranes well resolved, (2) the fact that all species examined show the same transition on the same section from PMFGM to SMFGM plus RPMFGM with the production of very similar paracrystalline organisation in the residual frequently finger-fringed RPMFGM areas in four of them and (3) the equivalent structures demonstrable in negatively stained and freeze-fractured specimens. It is also important to note that the PMFGM transition plus crystalline arrays can be found after using three very different primary fixatives: glutaraldehyde, osmium tetroxide or dichromate–acrolein.

The most likely proteins to interact to form the unit structure of the paracrystalline arrangements are BTN, XOR and ADPH (Ishii et al. 1995); Jeong et al. 2009; MacManaman et al. 2002; Chong et al. 2011). In addition, FRAP analysis showed that a fraction of endogenously expressed GFP-BTN was immobile in condensed areas on the surface of mouse MFGs, suggesting incorporation into a macromolecular complex (Jeong et al. 2013). The immunocytochemical results in this and our previous paper (Wooding and Sargeant 2015) clearly demonstrate the presence of BTN and XOR only in the PMFGM with ADPH throughout the SMFGM.

The nature of the apparently structureless areas of the SMFGM lacking an intact unit membrane is less certain. At a minimum, these regions will comprise the phospholipid monolayer covering the entire globule surface and be present on all cellular lipid droplets (Martin and Parton 2006), plus the PAT family protein ADPH (Chong et al. 2011; Jeong et al. 2013; Wooding and Sargeant 2015). There may be other proteins (Walther and Farese 2012), including elements of the outer phospholipid bilayer of the PMFGM, that were not incorporated into the paracrystalline regions.

Corroboration of the reality of the PMFGM to SMFGM plus RPMFGM transition is provided by the results from unfixed mouse MFGs from the endogenously GFP-expressing *mT/mG* strain. The GFP-fluorophore can be considered a marker for an intact bilayer membrane (Muzumdar et al. 2007). There is a striking equivalence between the confocal images of GFP in transverse sections and 3D reconstructions marking regions of the intact bilayer and the morphology of the RPMFGM areas identified on the electron micrographs. Furthermore, when a GFP-BTN fusion protein was expressed in mouse mammary gland using an adenoviral vector, a fraction of the endogenously produced protein condensed on the surfaces of secreted droplets in similar patterns (Jeong et al. 2013).

These CLSM results are very similar to those using exogenous fluorescent lipid or lectin probes to label the unfixed MFG surfaces from bovine or human milk (Evers et al. 2008; Gallier et al. 2010, 2015; Lopez et al. 2010; Lopez 2011; Zheng et al. 2013). The results with both markers show discontinuities (nonfluorescent microdomains) that divide the fluorescence into “patches and networks (Lopez et al. 2010). Lopez interprets these frequently circular nonfluorescent microdomains as local aggregates of molecules (e.g., sphingomyelin) that exclude the probes but with no immunocytochemical evidence for this as yet. Evers et al. (2008) considered the domains to indicate the lack of a unit membrane, which would agree with our TEM observations. Our serial reconstructions of MFGs show similar circular areas (Fig. 6) corresponding to the domains without unit membrane bound RPMFGM .

The uniformity of the detail of this PMFGM formation and transition to SMFGM plus RPMFGM in all species examined at sufficient resolution reinforces the idea that the unique lipid secretion mechanism and subsequent modification method evolved only once in the mammalian lineage. The argument that it is not possible to generalise about the mechanism of MFG secretion because only a small number of mammals have been examined (Heid and Keenan 2005) seems to be contradicted by the similarities in the detail now available in all metatherian and eutherian species adequately investigated.

## Abbreviations

ADPH: Adipohilin(Plin2)
BTN: Butyrophilin
CLSM: Confocal laser scanning microscopy
EM: Electron microscope/ic
FRAP: Fluorescence recovery after photobleaching
GFP: Green fluorescent protein
MFG: Milk fat globule
MFGM: Milk fat globule membrane
PMFGM: Primary milk fat globule membrane
RPMFGM: Residual primary milk fat globule membrane
SMFGM: Secondary milk fat globule membrane
TEM: Transmission electron microscopy
XOR: Xanthine oxidoreductase
